# Assessment of Cytotoxicity and Genotoxicity of Plasma-Treated Perfluorooctanesulfonate Containing Water Using In Vitro Bioassays

**DOI:** 10.3390/toxics12120889

**Published:** 2024-12-06

**Authors:** Markus Windisch, Roman Klymenko, Hannah Grießler, Clemens Kittinger

**Affiliations:** 1 Diagnostic and Research Institute of Hygiene, Microbiology and Environmental Medicine, Diagnostic and Research Center for Molecular Biomedicine, Medical University of Graz, 8010 Graz, Austria; markus.windisch@medunigraz.at (M.W.);; 2 Wetsus, European Centre of Excellence for Sustainable Water Technology, 8911 MA Leeuwarden, The Netherlands; roman.klymenko@wetsus.nl; 3 Electrical Energy Systems Group, Department of Electrical Engineering, Eindhoven University of Technology, 5600 MB Eindhoven, The Netherlands

**Keywords:** PFAS, plasma treatment, cytotoxicity, genotoxicity

## Abstract

The contamination of ground and surface waters with per- and polyfluoroalkyl substances (PFASs) is of major concern due to their potential adverse effects on human health. The carbon–fluorine bond makes these compounds extremely stable and hardly degradable by natural processes. Therefore, methods for PFAS removal from water are desperately needed. In this context, plasma treatment of water has been proposed as an effective method with reported removal rates exceeding 90%. However, the high reactivity of plasma discharge results in the formation of many reactive species, like radicals, ozone, or even solvated electrons, which lead to a complex reaction cascade and, consequently, to the generation of a wide variety of different chemical products. The toxicological properties of these PFAS breakdown products are largely unknown. The present study focuses on a toxicological assessment of PFAS-containing plasma-treated water samples. Aqueous solutions of long-chain perfluorooctanesulfonate (PFOS) were treated with various plasma-atmospheric regimes. Subsequently, plasma-treated water samples were subjected to in vitro bioassays. Cytotoxicity and genotoxicity were assessed with the MTS assay using human liver cells (HepG2) and the Ames MPF^TM^ assay using *Salmonella* Typhimurium strains. Our results demonstrate varying cyto- and genotoxic properties of water containing PFAS breakdown products depending on the atmosphere present during plasma treatment. Based on the results of this study, the atmosphere used during plasma treatment affects the toxicological properties of the treated sample. Further studies are therefore needed to uncover the toxicological implications of the different treatment parameters, including the PFAS starting compound, the atmosphere during treatment, as well as the quantity of plasma energy applied.

## 1. Introduction

Per- and polyfluorinated alkyl substances (PFASs) are ubiquitous in our daily lives. Due to their water-repellent properties, these substances have been used in a multitude of industrial and commercial applications. The carbon–fluorine bond is one of the strongest chemical bonds, which contributes to the extreme stability and bioaccumulation potential of PFASs. Several investigations have identified the presence of PFASs in the environment [1,2,3], as well as biological tissues and fluids [4,5,6]. Human exposure to PFASs has been associated with adverse health effects, including liver disease [7,8], cancer [9,10] and altered immune function [11].

Because drinking water represents a main route of exposure to PFASs for humans, efforts have been taken to reduce PFAS contamination in ground and surface waters. The successful removal of PFASs from water has been demonstrated by a number of methods, including ion exchange [12], adsorption [13], and nanofiltration [14]. However, the aforementioned processes are exclusively focused on the removal of PFASs, rather than their complete eradication. Consequently, the initial issue persists, as PFAS molecules remain in the ecosystem, necessitating the development of long-term storage solutions. In this context, the plasma-based water treatment process is a promising alternative. The use of gas discharge plasma in contaminated water treatment integrates several advanced techniques, including ultraviolet irradiation, advanced oxidation via reactive oxygen and nitrogen species (RONS), electrolysis, and shockwave-based water purification. This process also generates highly reactive solvated electrons (e^−^_(aq)_) in the gas–liquid interface, along with energetic electrons (e^−^) and ions (X^±^) within the plasma [15,16,17]. All of these effects contribute to the breakdown of PFAS molecules into shorter chains, but further degradation of these shorter chains is normally less effective [18,19]. Some authors [16,20] significantly improved the degradation of both long and short chains by adding a cationic surfactant to the reactor. The electrostatic forces cause PFASs and surfactant molecules to bind together, enabling the surfactant to transport PFASs closer to the plasma discharge, thereby enhancing degradation. Although this technology appears to have potential for the breakdown and removal of both long- and short-chain PFASs, there is currently limited knowledge regarding the toxicity of the generated breakdown products.

**Figure 1:**
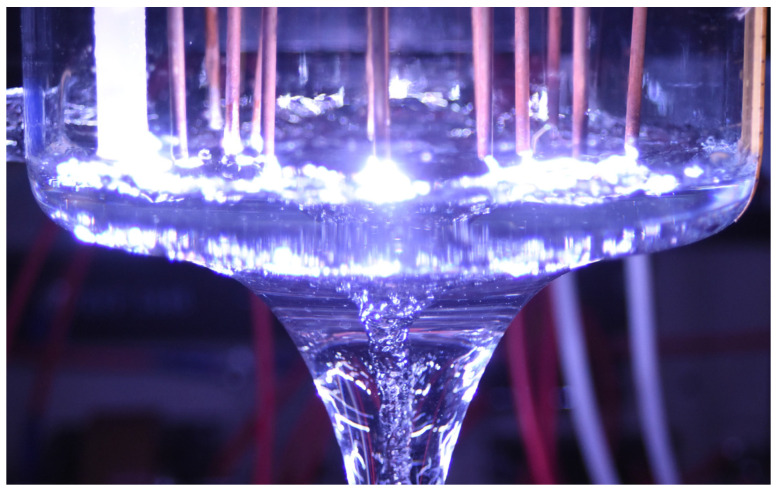
Hyperbolic vortex plasma reactor [17] during operation.

The objective of this study was to investigate the toxicity of PFAS degradation products upon treatment of water with a contact plasma reactor. In particular, reaction products from a novel hyperbolic vortex plasma reactor (Figure 1) were investigated [17]. In this reactor, the combination of plasma discharge with a Schauberger hyperbolic vortex [21,22] enables the on-site production of RONS in the gas phase via plasma discharge, while the water vortex ensures efficient mass transfer and mixing through secondary vortices, thus allowing the reactive species to be distributed into the liquid phase. This combined technique has been widely recognized for significantly improving the degradation of micropollutants [17]. Additionally, its unique electrode configuration creates a substantial plasma–water interface, leading to increased RONS production and enhanced degradation of micropollutants.

Perfluorooctanesulfonate (PFOS), a long-chain “legacy” PFAS, was used as a model substance for plasma treatment. The diverse breakdown products of PFOS upon plasma treatment have been previously described [15]. Various atmospheric conditions were tested during plasma treatment, which influenced the generation of reactive species. Subsequently, the plasma-treated water samples were subjected to in vitro bioassays. Given the established hepatotoxicity of PFASs, an assessment of cytotoxicity was conducted using the MTS assay with human liver cells (HepG2). HepG2 cells are a well-established model for in vitro toxicological investigations. They are capable of phase I enzymatic liver modification, thereby obviating the necessity of an external metabolic activation source. Furthermore, numerous studies have indicated that PFASs possess hepatotoxic properties. For these reasons, the HepG2 cell line was chosen in the present study for cytotoxicity evaluation of PFOS breakdown products after plasma treatment. To assess genotoxicity, the Ames-MPF™ assay was used. This is the first study investigating the toxicological properties of PFAS breakdown products after plasma treatment of PFAS-contaminated water.

## 2. Materials and Methods

### 2.1. Preparation, Treatment, and Analyses of the PFOS Sample

PFOS (40% in H_2_O, CAS No.:1763-23-1) was purchased from Sigma-Aldrich, Amsterdam, The Netherlands. PFOS solutions were prepared by spiking PFOS into deionized (DI) water. Then, the samples were treated for 75 min with 1.2 kWh/m^3^ of plasma power. More detailed information about the treatment process, setup, power supply, and energy measurements can be found elsewhere [17]. PFOS samples were collected at set time intervals and analyzed for concentrations using liquid chromatography–mass spectrometry (6420 triple Quad LC/MS, Agilent Technologies, Santa Clara, CA, USA). For the analysis, an Agilent Zorbax Eclipse Plus C18 RRHD column (1.8 μm, 50 × 2.1 mm) was used. The samples were diluted by 1:1 with methanol for stability, and 1 mL was pipetted into a plastic cation vial. For the toxicological assay, samples were concentrated 25× times using a rotary evaporator Buchi Rotavapor R-3 and the vacuum pump KNF NEUBERGER Freiburg D-79112. Detailed sample information can be found in the Supplemental Material (Table S1).

### 2.2. Cell Culture

Human liver cells (HepG2, DSMZ ACC 180) were maintained in Roswell Park Memorial Institute Medium (RPMI 1640, Merck, Wien, Austria) supplemented with 10% (*v*/*v*) fetal bovine serum (FBS, Merck, Austria). Cells were incubated in a 5% CO_2_-humidified incubator. The cells were subcultured twice a week using a 1:5 split ratio.

### 2.3. MTS Cell Viability Assay

The cytotoxicity of plasma-treated PFOS water samples was assessed using the MTS assay kit (CellTiter 96^®^ Aqueous One Solution Assay) purchased from Promega Corporation, Alexandria, Australia. The assay was carried out in accordance with the instructions provided by the manufacturer. Briefly, HepG2 cells were seeded at a density of 1 × 10^5^ cells per 90 µL in a 96-well flat clear bottom plate. After overnight incubation, cells were for exposed for 20 h to plasma-treated PFOS water samples at a concentration of 10, 5, 2.5, and 1.25% (*v*/*v*), respectively. After 4 h of incubation, 20 µL of the MTS reagent was added to each well. Absorbance was measured at a wavelength of 492 nm. The cytotoxic effect of plasma-treated PFOS water samples on HepG2 cells was calculated using Equation (1).

(1) $$\mathrm{Cell}\;\mathrm{viability}=\frac{100\times Abs492\;sample}{Abs492\;control}$$

### 2.4. Ames MPF Assay

The Ames-MPF^TM^ Penta 1 assay kit was purchased from Xenometrix (Allschwil, Switzerland) and conducted in accordance with the instructions provided by the manufacturer. *Salmonella* Typhimurium strains TA 98 and TA 100 were used because this strain combination already detects 93% of genotoxins [23]. Briefly, *Salmonella* Typhimurium TA 98 and TA 100 cultures were grown overnight in 10 mL of Nutrient Broth No. 2 (Oxoid, Basingstoke, Hampshire, UK) supplemented with 10 µL of Ampicillin solution prepared from 50 mg of Ampicillin sodium salt (Carl Roth, Karlsruhe, Germany) dissolved in 1 mL of deionized water. Only cultures that reached an OD_600_ value of at least 2 were used for the testing procedure. Then, 10 µL of each 25× concentrated plasma-treated PFOS water sample was mixed with 240 µL of overnight culture suspended in exposure medium (10% (*v*/*v*) TA 98, 5% (*v*/*v*) TA 100) in 24-well plates. Bacterial cells were exposed to the samples for 90 min at 37 °C and 240 rpm shaking. After incubation, 2.65 mL of reversion indicator medium was added to each well. Each sample was plated in 384-well plates (triplicates) and incubated for 48 h at 37 °C. After incubation, yellow revertant wells were scored. Every sample was tested with and without the presence of an external metabolic activation source (S9 fraction). A detailed description of the test procedure with metabolic activation can be found in the manufacturer’s manual.

#### Data Evaluation and Statistical Analysis

Ames MPF^TM^ data analysis was conducted according to the method recommended by Xenometrix [24]. Briefly, for each tested dose the mean number of positive wells from the triplicates was calculated, and the fold increase over the baseline (mean of the negative control plus 1 SD) was determined. Additionally, all doses were tested for statistical significance with Student’s *t*-test against the negative control. If a sample yields multiple fold inductions greater than 2.0 as well as at least two adjacent doses with *p* < 0.05, it is considered mutagenic.

For the assessment of cytotoxicity, the reduction of positive revertant wells was considered as well as the increased brilliance of the purple reversion medium due to bacterial lysis or lack of growth.

## 3. Results

### 3.1. PFAS Concentrations

**Figure 2:**
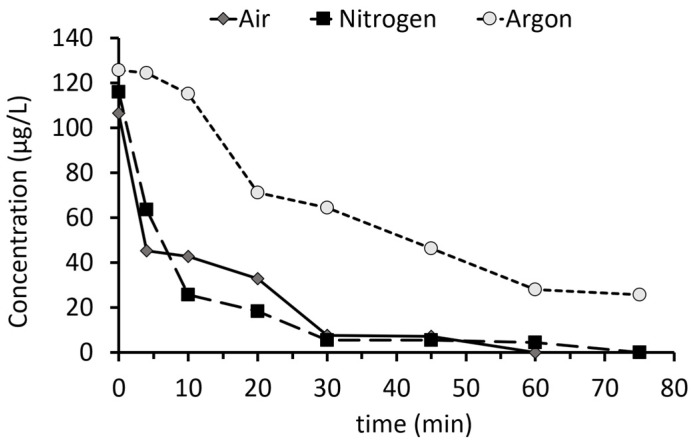
PFOS degradation over time in hyperbolic vortex plasma reactor for three gas compositions: air, nitrogen, and argon.

Figure 2 illustrates PFOS degradation over time across the three atmospheres tested: air, nitrogen, and argon. The most rapid degradation was observed in air, with the PFOS concentration falling below the detection limit after 60 min. Under the nitrogen atmosphere, PFOS was no longer detectable after 75 min. In contrast, under argon, the PFOS concentration remained above 25 μg/L even after 75 min of treatment.

**Figure 3:**
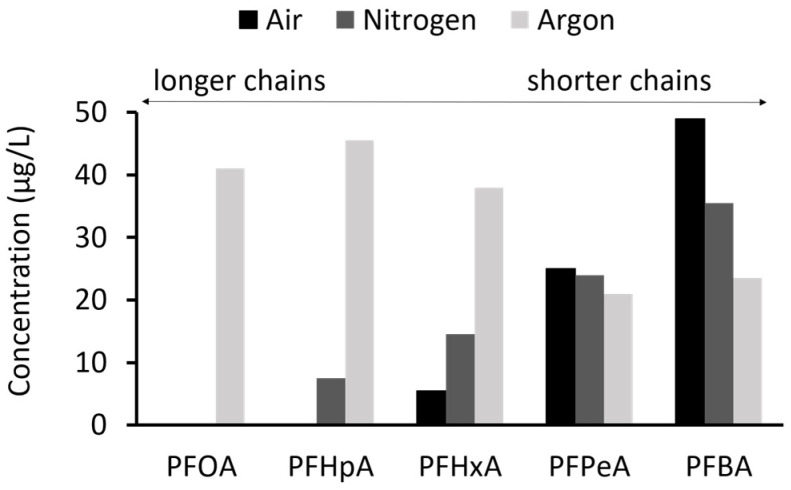
Degradation products of PFOS after treatment in hyperbolic vortex plasma reactor in concentrated samples for three gas compositions: air, nitrogen, and argon. Perfluorooctanoic acid (PFOA); perfluoroheptanoic acid (PFHpA); perfluorohexanoic acid (PFHxA); perfluoropentanoic acid (PFPeA); and perfluorobutanoic acid (PFBA).

Figure 3 shows the degradation products of PFOS after treatment in concentrated samples. A higher concentration of longer chains was detected in the argon atmosphere, whereas the air atmosphere resulted in a higher concentration of shorter chains, with concentrations for the nitrogen atmosphere falling between these two. Notably, the longest chain degradation product, PFOA, was observed exclusively in the argon atmosphere.

### 3.2. MTS Results

**Figure 4:**
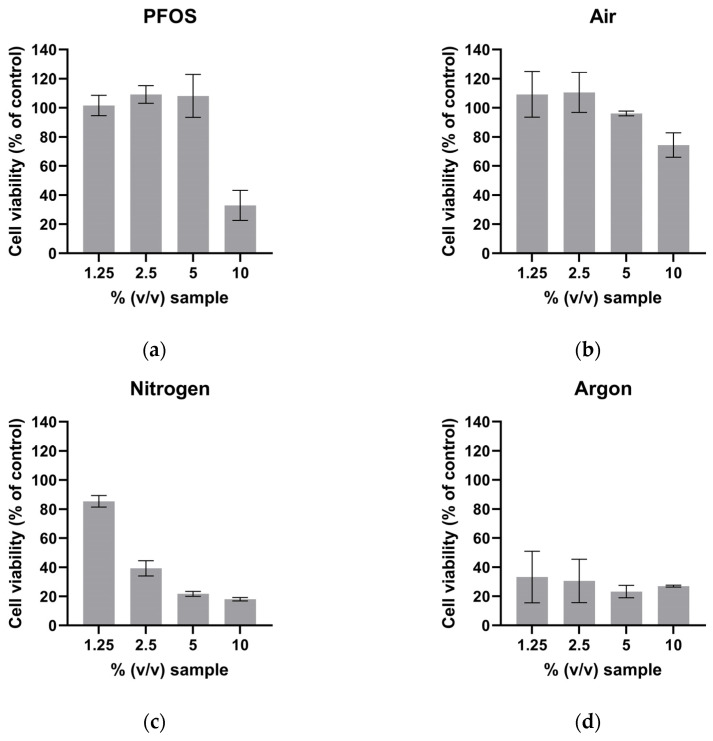
MTS results of PFOS-containing water after plasma treatment with different atmospheres. (**a**) Untreated PFOS control, (**b**) treatment with ambient air, (**c**) treatment with nitrogen, (**d**) treatment with argon. Error bars represent the standard deviation of three replicates.

The MTS test demonstrated a dose-dependent cytotoxic effect for all investigated samples (Figure 4). The sample treated under an argon atmosphere (Figure 4d) exhibited pronounced cytotoxicity even at the lowest concentration tested (1.25% *v*/*v*). The sample plasma-treated under ambient air atmosphere (Figure 4b) demonstrated the lowest degree of cytotoxicity among all tested samples at a concentration of 10% (*v*/*v*).

### 3.3. Ames MPF^TM^ Results

**Figure 5:**
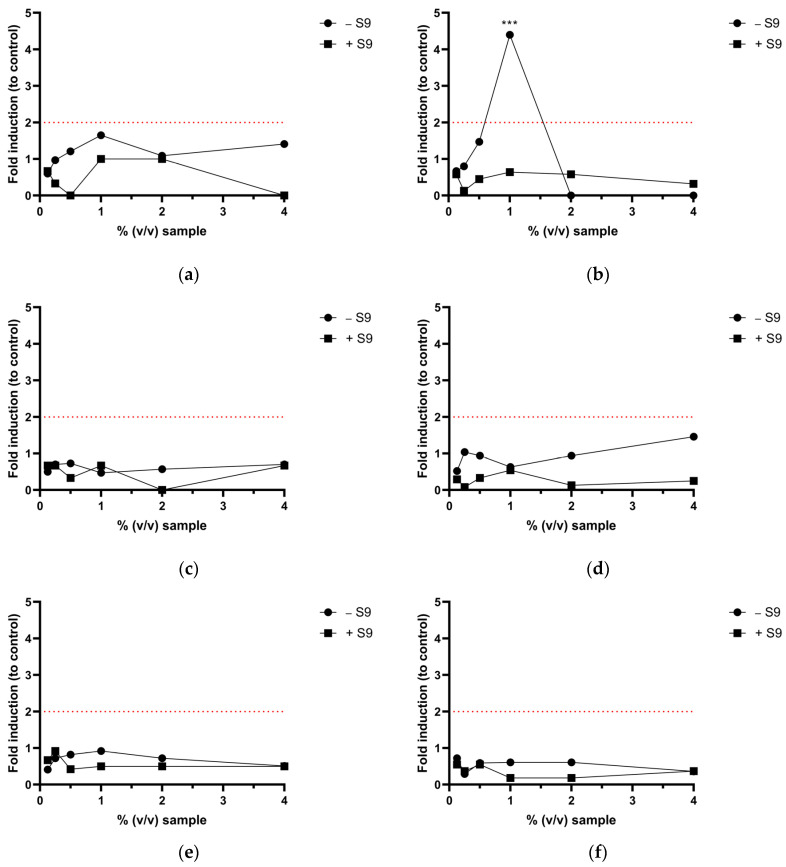

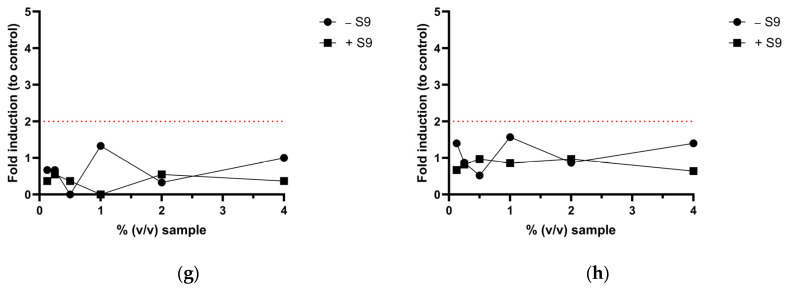
Results of the Ames MPF^TM^ assay of PFOS-containing water after plasma treatment with different atmospheres. The red dotted line represents a 2-fold increase over baseline. A fold increase over the baseline of ≥2.0 is a positive response in the Ames MPF^TM^ test. (**a**) Argon atmosphere; *S.* Typhimurium TA 98 w./w.o. S9 mix. (**b**) Argon atmosphere; *S.* Typhimurium TA 100 w./w.o. S9 mix. (**c**) Nitrogen atmosphere; *S.* Typhimurium TA 98 w./w.o. S9 mix. (**d**) Nitrogen atmosphere; *S.* Typhimurium TA 100 w./w.o. S9 mix. (**e**) Ambient air; *S.* Typhimurium TA 98 w./w.o. S9 mix. (**f**) Ambient air; *S.* Typhimurium TA 100 w./w.o. S9 mix. (**g**) Untreated PFOS control; *S.* Typhimurium TA 98 w./w.o. S9 mix. (**h**) Untreated PFOS control; *S.* Typhimurium TA 100 w./w.o. S9 mix. *t*-test: *** *p* < 0.001.

The Ames MPF^TM^ assay revealed a genotoxic signal in the sample plasma-treated with argon atmosphere (Figure 5b). This finding was exclusive for this condition as no other plasma-atmospheric regime induced genotoxicity.

### 3.4. Dose-Dependent Genotoxicity of Plasma Treated, PFOS Containing Water Under Argon Atmosphere

**Figure 6:**
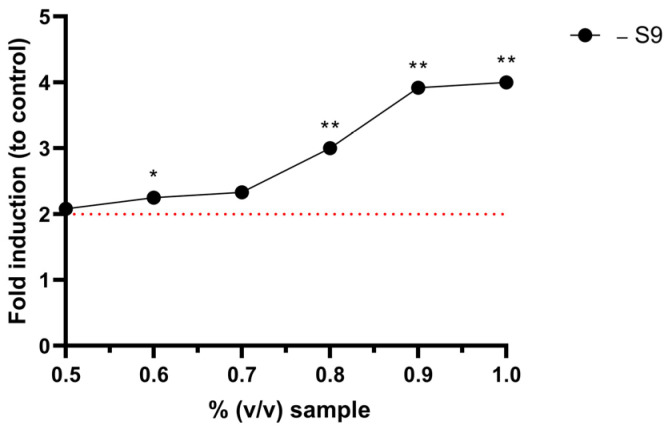
Ames MPF^TM^ (*S.* Typhimurium TA 100, w.o. S9 mix) results of PFOS-containing water plasma-treated with argon atmosphere with a modified dose-range of 1 to 0.5% (*v*/*v*). The red dotted line represents 2-fold over baseline. A fold increase over baseline of ≥2.0 is a positive response in the Ames MPF^TM^ test. *t*-test: * *p* < 0.05; ** *p* < 0.01.

For the initial Ames MPF^TM^ test, a 1:1 serial dilution of the sample was carried out according to manufacturer’s instructions. The six concentrations tested ranged from 4% (*v*/*v*) to 0.125% (*v*/*v*). However, in this experimental setup, the plasma-treated sample with argon atmosphere exhibited a fold increase greater than 2 only at a concentration of 1% (*v*/*v*). At higher concentrations, the cytotoxicity of the sample inhibited the induction of genotoxicity. Therefore, a smaller dose range was selected for subsequent testing. The modified test setup yielded a clear dose–response relationship between the sample concentration and the revertant count (Figure 6). This is indicative of a true genotoxic effect.

## 4. Discussion

Plasma treatment successfully broke down PFOS, a legacy compound, into long- and short-chained PFASs, specifically PFOA, PFHpA, PFHxA, PFPeA, and PFBA. The specific PFOS degradation products formed are contingent upon the atmosphere utilized during the plasma treatment process. Plasma treatment under argon atmosphere biased the formation of longer-chained PFASs, while treatment with air and nitrogen biased the formation of shorter-chains. PFOA, a long-chain legacy PFAS, was exclusively detectable in the sample plasma-treated under argon atmosphere. Two standard in vitro toxicity assays were used to assess the efficacy of the plasma technologies in reducing cytotoxicity and mutagenicity.

Our results demonstrate that the composition of the atmosphere during the plasma treatment affects the cytotoxicity of the sample. The plasma treatment of PFOS-containing water under an argon atmosphere resulted in the highest cytotoxicity. However, treatment under ambient air resulted in overall low cytotoxicity, comparable to that of the untreated PFOS control. These differences are probably due to more reactive oxygen species generated in the argon atmosphere. The formation of predominantly reactive nitrogen species (RNS) is observed when the treatment is conducted under nitrogen, whereas the formation of reactive oxygen species (ROS) is observed when the treatment is conducted under argon [17].

Single PFASs are generally regarded as non-genotoxic. Accordingly, in this study, untreated aqueous PFOS solution was demonstrated to be non-genotoxic in the Ames MPF^TM^ assay at the concentrations tested. However, a study conducted by Ojo et al. demonstrated the genotoxicity of PFAS mixtures when tested with the comet assay but without a dose–response relationship [25]. In this study, we report for the first time in vitro genotoxicity of a PFAS mixture when tested with the Ames test. Additionally, a dose–response relationship was found. Plasma treatment with argon atmosphere resulted in a genotoxic signal in *S.* Typhimurium TA 100 without metabolic activation. The effect was dose-dependent, leading to an over 4-fold increase over the baseline (Figure 5b). Additionally, when a smaller dose range was tested, three adjacent doses displayed a fold increase greater than two and *p* < 0.01 when tested with Student’s *t*-test (Figure 6). There have been no reports in the scientific literature to date on Ames-positive PFASs. However, the chemical processes involved in the breakdown of PFOS during plasma treatment are complex. In addition to shorter-chain PFASs, the formation of fluoride ions, smaller organic acids, and ROS/RNS has been described [15]. Thus, it cannot be concluded with certainty that the genotoxic effect stems from shorter chain PFASs. Other compounds or a mixture effect may play a role. The reason why genotoxicity occurs exclusively in plasma treatment under argon atmosphere could not be resolved during this study. However, as shown in Figure 3, the concentrations of long-chain degradation products formed in the argon atmosphere are significantly higher than those in nitrogen or air atmospheres. Additionally, the concentration of short-chain PFASs formed in the argon atmosphere is lower than in the other two. Assuming that shorter chains are generally less toxic than longer chains [26] we hypothesize that the toxicity of the argon-treated samples could be partially attributed to the presence of more long-chained PFAS molecules compared to those formed in other atmospheres.

## 5. Conclusions

The development of safe and effective methods to remove PFASs from ground and surface waters is important for humans and the environment. This study demonstrates the efficacy of combining a hyperbolic vortex with plasma treatment for breakdown of the legacy compound PFOS in water. The formation of PFOS breakdown products and the cyto- and genotoxicity of the treated water were found to be affected by different plasma-atmospheric regimes. Because PFASs comprise a large group of fluorinated chemicals, a wider variety of compounds should be tested in the course of further studies. The investigations presented here represent merely the initial phase in the characterization of the degradation products, with further and more complex investigations employing in vivo models required to reflect chronic adverse health effects (e.g., cancer, hyperlipidemia, thyroid dysfunction, or immune suppression).

## Data Availability

The data will be made available upon request, which should be sent to markus.windisch@medunigraz.at. Requests are subject to approval by all of the named authors participating in this study/the Supplementary Materials.

## Supplementary Materials

The following supporting information can be downloaded at https://www.mdpi.com/article/10.3390/toxics12120889/s1, Table S1: Concentrations of PFAS species in plasma-treated water samples.

## References

[1] Müller V., Kindness A., Feldmann J. (2023). Fluorine Mass Balance Analysis of PFAS in Communal Waters at a Wastewater Plant from Austria. Water Res..

[2] Moody C.A., Field J.A. (1999). Determination of Perfluorocarboxylates in Groundwater Impacted by Fire-Fighting Activity. Environ. Sci. Technol..

[3] Kwok K.Y., Yamazaki E., Yamashita N., Taniyasu S., Murphy M.B., Horii Y., Petrick G., Kallerborn R., Kannan K., Murano K. (2013). Transport of Perfluoroalkyl Substances (PFAS) from an Arctic Glacier to Downstream Locations: Implications for Sources. Sci. Total Environ..

[4] Sunderland E.M., Hu X.C., Dassuncao C., Tokranov A.K., Wagner C.C., Allen J.G. (2019). A Review of the Pathways of Human Exposure to Poly- and Perfluoroalkyl Substances (PFASs) and Present Understanding of Health Effects. J. Expo. Sci. Environ. Epidemiol..

[5] Hall S.M., Zhang S., Hoffman K., Miranda M.L., Stapleton H.M. (2022). Concentrations of Per- and Polyfluoroalkyl Substances (PFAS) in Human Placental Tissues and Associations with Birth Outcomes. Chemosphere.

[6] Worley R.R., Moore S.M.A., Tierney B.C., Ye X., Calafat A.M., Campbell S., Woudneh M.B., Fisher J. (2017). Per- and Polyfluoroalkyl Substances in Human Serum and Urine Samples from a Residentially Exposed Community. Environ. Int..

[7] Zhang X., Zhao L., Ducatman A., Deng C., von Stackelberg K.E., Danford C.J., Zhang X. (2023). Association of Per- and Polyfluoroalkyl Substance Exposure with Fatty Liver Disease Risk in US Adults. JHEP Rep..

[8] Nian M., Li Q.Q., Bloom M., Qian Z., Syberg K.M., Vaughn M.G., Wang S.Q., Wei Q., Zeeshan M., Gurram N. (2019). Liver Function Biomarkers Disorder Is Associated with Exposure to Perfluoroalkyl Acids in Adults: Isomers of C8 Health Project in China. Environ. Res..

[9] Mastrantonio M., Bai E., Uccelli R., Cordiano V., Screpanti A., Crosignani P. (2018). Drinking Water Contamination from Perfluoroalkyl Substances (PFAS): An Ecological Mortality Study in the Veneto Region, Italy. Eur. J. Public Health.

[10] Messmer M.F., Salloway J., Shara N., Locwin B., Harvey M.W., Traviss N. (2022). Risk of Cancer in a Community Exposed to Per- and Poly-Fluoroalkyl Substances. Environ. Health Insights.

[11] Goudarzi H., Miyashita C., Okada E., Kashino I., Chen C.J., Ito S., Araki A., Kobayashi S., Matsuura H., Kishi R. (2017). Prenatal Exposure to Perfluoroalkyl Acids and Prevalence of Infectious Diseases up to 4 Years of Age. Environ. Int..

[12] Dixit F., Barbeau B., Mostafavi S.G., Mohseni M. (2019). PFOA and PFOS Removal by Ion Exchange for Water Reuse and Drinking Applications: Role of Organic Matter Characteristics. Environ. Sci..

[13] Appleman T.D., Higgins C.P., Quiñones O., Vanderford B.J., Kolstad C., Zeigler-Holady J.C., Dickenson E.R.V. (2014). Treatment of Poly- and Perfluoroalkyl Substances in U.S. Full-Scale Water Treatment Systems. Water Res..

[14] Steinle-Darling E., Reinhard M. (2008). Nanofiltration for Trace Organic Contaminant Removal: Structure, Solution, and Membrane Fouling Effects on the Rejection of Perfluorochemicals. Environ. Sci. Technol..

[15] Singh R.K., Fernando S., Baygi S.F., Multari N., Thagard S.M., Holsen T.M. (2019). Breakdown Products from Perfluorinated Alkyl Substances (PFAS) Degradation in a Plasma-Based Water Treatment Process. Environ. Sci. Technol..

[16] Nau-Hix C., Multari N., Singh R.K., Richardson S., Kulkarni P., Anderson R.H., Holsen T.M., Thagard S.M. (2021). Field Demonstration of a Pilot-Scale Plasma Reactor for the Rapid Removal of Poly- and Perfluoroalkyl Substances in Groundwater. ACS EST Water.

[17] Klymenko R., de Kroon E., Agostinho L.L.F., Fuchs E.C., Woisetschläger J., Hoeben W.F.L.M. (2024). Characterization of a Hyperbolic Vortex Plasma Reactor for the Removal of Aqueous Phase Micropollutants. J. Phys. D Appl. Phys..

[18] Palma D., Richard C., Minella M. (2022). State of the Art and Perspectives about Non-Thermal Plasma Applications for the Removal of PFAS in Water. Chem. Eng. J. Adv..

[19] Jiang L., Wang S., Chen W., Lin J., Yu X., Feng M., Wan K. (2022). Removal of Per-and Polyfluoroalkyl Substances by Electron Beam and Plasma Irradiation: A Mini-Review. Water.

[20] Isowamwen O., Li R., Holsen T., Thagard S.M. (2023). Plasma-Assisted Degradation of a Short-Chain Perfluoroalkyl Substance (PFAS): Perfluorobutane Sulfonate (PFBS). J. Hazard. Mater..

[21] Donepudi T., van de Griend M., Agostinho L.L.F., de Kroon E.J., Klymenko R., Pecnik R., Woisetschläger J., Fuchs E.C. (2024). Numerical Analysis of Vortex Dynamics in Hyperbolic Funnels Using Computational Fluid Dynamics. Phys. Fluids.

[22] Klymenko R., Nanninga H., de Kroon E., Agostinho L.L.F., Fuchs E.C., Woisetschläger J., Hoeben W.F.L.M. (2023). Preparation of Free-Surface Hyperbolic Water Vortices. J. Vis. Exp..

[23] Williams R.V., DeMarini D.M., Stankowski L.F., Escobar P.A., Zeiger E., Howe J., Elespuru R., Cross K.P. (2019). Are All Bacterial Strains Required by OECD Mutagenicity Test Guideline TG471 Needed?. Mutat. Res. Genet. Toxicol. Environ. Mutagen..

[24] Flückiger-Isler S., Kamber M. (2012). Direct Comparison of the Ames Microplate Format (MPF) Test in Liquid Medium with the Standard Ames Pre-Incubation Assay on Agar Plates by Use of Equivocal to Weakly Positive Test Compounds. Mutat. Res. Genet. Toxicol. Environ. Mutagen..

[25] Ojo A.F., Peng C., Ng J.C. (2022). Genotoxicity Assessment of Per- and Polyfluoroalkyl Substances Mixtures in Human Liver Cells (HepG2). Toxicology.

[26] Ojo A.F., Peng C., Ng J.C. (2020). Combined Effects and Toxicological Interactions of Perfluoroalkyl and Polyfluoroalkyl Substances Mixtures in Human Liver Cells (HepG2). Environ. Pollut..

